# Sarcopenia for predicting falls and hospitalization in community-dwelling older adults: EWGSOP versus EWGSOP2

**DOI:** 10.1038/s41598-019-53522-6

**Published:** 2019-11-27

**Authors:** Ming Yang, Ying Liu, Yun Zuo, Huairong Tang

**Affiliations:** 1 The Center of Gerontology and Geriatrics, West China Hospital, Sichuan University, No. 37 Guoxue Lane, Chengdu, Sichuan China; 2 Precision Medicine Research Center, West China Hospital, Sichuan University, No. 37 Guoxue Lane, Chengdu, Sichuan China; 3 Health Management Center, Shangjin Nanfu Hospital, Chengdu, Sichuan China; 4 Health Management Center, West China Hospital, Sichuan University, No. 37 Guoxue Lane, Chengdu, Sichuan China

**Keywords:** Geriatrics, Prognosis

## Abstract

The European Working Group on Sarcopenia in Older People (EWGSOP) recently published an updated version (EWGSOP2). We aimed to compare the predictive values of EWGSOP-defined and EWGSOP2-defined sarcopenia for the incidence of falls and hospitalization in older adults. We defined sarcopenia according to the EWGSOP and the EWGSOP2. We further modified the cut-off points of the EWGSOP and EWGSOP2 according to the lowest quintile values of the gender-specific distribution of our study population, named “modified EWGSOP” and “modified EWGSOP2”, respectively. We included 384 participants. During the follow-up, 98 participants (26.5%) and 51 participants (13.8%) had at least one fall or hospitalization, respectively. EWGSOP2-defined sarcopenia (hazard ratio [HR] 1.86, 95% confidence interval [CI] 1.22–1.84) and modified EWGSOP2-defined sarcopenia (HR 2.09, 95% CI 1.23–3.55) were significantly associated with an increased incidence of falls, respectively. EWGSOP-defined sarcopenia and modified EWGSOP-defined sarcopenia also have a trend to be associated with the incidence of falls, but the results were not statistically significant. Only modified EWGSOP2-defined sarcopenia (HR 2.07, 95% CI 1.01–4.27) was significantly related to an increased incidence of hospitalization. In conclusion, EWGSOP2-defined sarcopenia performed more sensitive than EWGSOP-defined sarcopenia for predicting the incidence of falls or hospitalization, especially when using the modified cutoffs.

## Introduction

Sarcopenia refers to the loss of skeletal muscle mass (SMM) and muscle function^1^. Sarcopenia is traditionally treated as a geriatric syndrome^1^, but it has recently been recognized as a disease because it has gained an International Statistical Classification of Diseases and Related Health Problems (ICD) code (ICD-10-CM M62.84) since 2016^2^. Sarcopenia is currently an important research area not only in gerontology and geriatrics but also increasingly in other specialties, such as respiratory medicine and cardiology^3^ and public health^4^.

One major obstacle for sarcopenia research and its translation into clinical practice is the lack of a unique definition or diagnostic criteria of sarcopenia. To date, at least seven international groups have published their guidelines or consensuses for sarcopenia^5,6^. However, the European Working Group on Sarcopenia in Older People (EWGSOP) guideline is the first and the most widely used guideline in the research area of sarcopenia worldwide^3,7^. To date, the EWGSOP guideline has been cited in 3,484 papers (including 2,597 original articles) in the Web of Science database. Among these original articles, 851 were conducted in East Asia, including China. According to the EWGSOP, sarcopenia is defined by low SMM and low muscle strength (such as handgrip strength, HS), and/or low physical performance (such as gait speed, GS)^7^. EWGSOP-defined sarcopenia has been related to a number of adverse clinical outcomes, such as falls, fractures, physical frailty and disability, poor quality of life, and even death^8,9^.

Most recently, the EWGSOP published an updated version (EWGSOP2)^10^. According to the EWGSOP2, sarcopenia is defined only based on low SMM and low muscle strength, whereas low physical performance (such as GS) is no longer a component of sarcopenia but an option to determine the severity of sarcopenia^10^. Moreover, the cut-off points of low SMM and low HS are also altered in the EWGSOP2 (but the cutoff of low GS in the EWGSOP2 is the same as that in the EWGSOP)^7,10,11^. Due to these alterations, EWGSOP-defined and EWGSOP2-defined sarcopenia are theoretically different. However, the newer is not necessarily better. The EWGSOP2 needs to be validated in not only research but also in clinical practice. We, therefore, conducted a prospective study to compare the predictive values of EWGSOP-defined and EWGSOP2-defined sarcopenia for the incidence of falls and hospitalization in community-dwelling older adults.

## Methods

We conducted a prospective observational study named “Sarcopenia among Older Adults in Chengdu” (SOAC) study from October 2017 to November 2018. All participants (or their legal proxies for those who were unable to write their names) signed a written informed consent form. The Research Ethics Committee of West China Hospital, Sichuan University approved the study protocol. All the methods in this study were in line with relevant guidelines and regulations.

### Study population

The baseline investigation was conducted in October and November 2017. We continuously recruited older adults (aged 60 years and older) who lived in Shangjin Community in Chengdu, China. We excluded individuals with the following conditions: (1) with an implanted pacemaker; (2) clinically visible edema; (3) unable to walk due to any reason; (4) unable to talk to interviewers; (5) severe mental illness; (6) severe renal failure; (7) severe heart failure^12^. Trained nurses performed the face-to-face interviews, anthropometric measurements, the GS test, and the HS test.

### Body composition measurement

We measured the body fat mass and appendicular skeletal muscle mass (ASM) for each individual using a bioimpedance analysis (BIA) device (InBody 230, Biospace Co. Ltd., Korea). Before test, the participants were asked (1) to avoid alcohol or caffeinated beverages the night before their test; (2) to avoid eating or drinking within 5 hours of the test; (3) to avoid exercise within 6 hours of the test; (4) to urinate within 30 minutes of the test; and (5) to maintain normal body hydration. Next, we calculated the appendicular skeletal muscle mass index (ASMI) according to the equation: ASMI (kg/m^2^) = ASM/height^2^.

### HS measurement

We measured the HS of each participant using a handheld dynamometer based on strain gauge sensors (EH101, Xiangshan Inc., Guangdong, China). Each hand was tested three times, and the highest value was applied for the statistical analysis.

### GS measurement

We measured the GS using a 4-meter walking test. The participants started from the standing position and were asked to walk a 4-meter course at a usual pace without slowing down before the 4-meter line. Using a stopwatch, a trained experimenter recorded the consuming time to the nearest of 0.1 seconds from the moment the first foot had passed the starting line to the moment the first foot had passed the 4-meter line. GS was calculated using 4 meters divided by the elapsed time to the nearest of 0.1 m/s.

### Sarcopenia definitions

We defined sarcopenia according to the EWGSOP and the EWGSOP2, respectively. In addition, the EWGSOP and EWGSOP2 “focus on European populations”^10^ and the cut-off points of SMI, GS, and HS recommended by the EWGSOP and EWGSOP2 may not be suitable for the Chinese population. Therefore, we further modified the cut-off points of SMI, GS, and HS of the EWGSOP and EWGSOP2 according to the lowest quintile values of the gender-specific distribution of our study population as recommended previously^13–15^, named “modified EWGSOP” and “modified EWGSOP2” in this study, respectively. The detailed diagnostic criteria and the cut-off points of each component of sarcopenia are presented in Supplementary Table 1.

### Covariates

We collected the following information through face-to-face interviews: age, gender, hypertension, coronary heart disease, cognitive impairment, diabetes, stroke, chronic obstructive pulmonary disease, and history of falls in the previous year.

### Follow-up

At six and 12 months after the baseline investigation, we asked each participant the following questions via telephone interviews: “Have you fallen in the past six months? If yes, when did you fall?” and “Have you been hospitalized for any reason in the past six months? If yes, when were you hospitalized?” Falls were defined as “unintentionally coming to rest on the ground, floor, or other lower-level”^16^. The hospitalization information was further confirmed according to the local database of hospital records.

### Statistical analyses

We investigated the normality of continuous data using the Kolmogorov–Smirnov test. Then, we presented descriptive statistics as percentages or mean values and standard deviations (SD). We applied ANOVA and chi-square tests to compare the differences between groups for continuous data and categorical data, respectively.

We applied Cox proportional hazard models to calculate the hazard ratios (HRs) and 95% confidence intervals (CIs) of different sarcopenia definitions for predicting the incidence of falls and the incidence of hospitalization. In addition to the unadjusted model, we adjusted for age and gender in Model 1. In Model 2, we adjusted for age, gender, history of falls, and the covariates that had potential association with fall (p < 0.1) for the incidence of falls and adjusted for age, gender, and the covariates that had potential association with hospitalization (p < 0.1) for the incidence of hospitalization. In these models, we treated age as continuous data and the other covariates as categorical data. We further applied the Kaplan–Meier method to estimate the Kaplan–Meier curves and applied the log-rank test to compare the difference between these curves. Moreover, we compared the area under the receiver-operating characteristic curves (AUC) and 95% CI using the Delong method^17^.

We applied SPSS version 20.0 (SPSS Statistics; IBM, Armonk, NY) and MedCalc Statistical Software version 15.2 (MedCalc Software bvba, Ostend, Belgium) to perform the statistical analyses. A 2-tailed P value of <0.05 indicates statistically significant.

## Results

### Characteristics of participants

We included 384 participants (71.5 ± 5.8 years, 160 men and 224 women) in the baseline investigation. Fourteen participants (3.6%) were lost during the one-year follow-up. Compared to the participants who completed the study, those who were lost to follow-up were older (74.4 versus 71.4 years, p = 0.061) and were more prone to cognitive impairment (14.3% versus 3.2%, p = 0.030).

Table 1 shows the characteristics of the participants. Compared to the participants without falls, those with at least one fall were significantly older and more prone to cognitive impairment and coronary heart disease (Table 1). Compared to the participants without hospitalization, those with at least one hospitalization were significantly older and were more prone to hypertension (Table 1).

**Table 1:** Characteristics of the study population.

Characteristic	Baseline (n = 384)	Follow-up^a^			Follow-up^a^		
		Nonfaller (n = 272)	faller (n = 98)	p	Individuals without hospitalization (n = 319)	Individuals with at least one hospitalization (n = 51)	p
Age (years)^b^	71.5 (5.8)	70.6 (5.2)	73.6 (6.4)	<0.001	71.1 (5.7)	73.4 (5.2)	0.008
Women (%)	224 (58.3)	154 (56.6)	63 (64.3)	0.186	187 (58.6)	30 (58.8)	0.978
Comorbidities (%)							
Hypertension	116 (30.2)	78 (28.7)	36 (36.7)	0.139	89 (27.9)	25 (49.0)	0.002
Coronary heart disease	36 (9.4)	21 (7.7)	14 (14.3)	0.057	29 (9.1)	6 (11.8)	0.545
Diabetes	36 (9.4)	25 (9.2)	10 (10.2)	0.769	29 (9.1)	6 (11.8)	0.545
Stroke	47 (12.2)	34 (12.5)	13 (13.3)	0.845	37 (11.6)	10 (19.6)	0.111
COPD	32 (8.3)	21 (7.2)	11 (11.2)	0.290	27 (8.5)	5 (9.8)	0.752
Cognitive impairment	14 (3.6)	5 (1.8)	7 (7.1)	0.011	9 (2.8)	3 (5.9)	0.252
History of falls (%)	59 (15.4)	38 (14.0)	18 (18.4)	0.298	47 (14.7)	9 (17.6)	0.590
BMI (men, kg/m2)^b^	24.1 (3.3)	24.5 (3.3)	23.2 (3.2)	0.035	24.3 (3.3)	24.0 (3.3)	0.755
BMI (women, kg/m2)^b^	24.3 (3.3)	24.4 (3.2)	23.9 (3.4)	0.355	24.3 (3.3)	23.7 (2.9)	0.299
GS (men, m/s)^b^	0.9 (0.3)	0.9 (0.3)	0.9 (0.2)	0.522	0.9 (0.2)	1.0 (0.3)	0.365
GS (women, m/s)^b^	0.9 (0.2)	0.9 (0.2)	0.8 (0.2)	<0.001	0.9 (0.2)	0.8 (0.2)	0.015
HS (men, kg)^b^	29.4 (8.9)	29.7 (9.0)	28.7 (9.1)	0.578	29.8 (9.1)	27.2 (8.2)	0.222
HS (women, kg)^b^	18.3 (5.4)	19.3 (5.2)	16.2 (5.2)	<0.001	18.8 (5.3)	16.0 (5.1)	0.009
ASM men, (kg)^b^	18.2 (3.0)	18.5 (2.8)	17.7 (3.5)	0.163	18.4 (3.0)	17.6 (2.6)	0.243
ASM (women, kg)^b^	12.6 (2.2)	12.8 (2.2)	12.0 (2.2)	0.018	12.7 (2.3)	12.1 (1.9)	0.182
Body fat mass (men, kg)^b^	18.1 (6.1)	18.7 (6.1)	16.9 (5.9)	0.121	18.2 (6.1)	18.7 (6.0)	0.742
Body fat mass (women, kg)^b^	19.6 (5.3)	19.7 (5.2)	19.2 (5.5)	0.518	19.7 (5.2)	18.4 (5.5)	0.196
EWGSOP-defined sarcopenia (%)	105 (27.3)	61 (22.4)	38 (38.8)	0.002	78 (24.5)	21 (41.2)	0.012
EWGSOP2-defined sarcopenia (%)	103 (26.8)	55 (20.2)	42 (42.9)	<0.001	77 (24.1)	20 (39.2)	0.023
Modified EWGSOP-defined sarcopenia (%)	45 (11.7)	22 (8.1)	20 (20.4)	0.001	31 (9.7)	11 (21.6)	0.013
Modified EWGSOP2-defined sarcopenia (%)	38 (9.9)	17 (6.3)	20 (20.4)	<0.001	27 (8.5)	10 (19.6)	0.014

^a.^Fourteen participants lost to follow-up during the 1-year follow-up.

^b.^Data are presented as the mean (standard deviation).

The Chi-square test was performed for categorical data and the ANOVA for continuous data. P < 0.05 indicates statistical significance.

ASM: appendicular skeletal muscle; BMI: body mass index; COPD: chronic obstructive pulmonary disease; EWGSOP: European Working Group on Sarcopenia in Older People; EWGSOP2: the updated version of the European Working Group on Sarcopenia in Older People; GS: gait speed; HS: handgrip strength.

### Prevalence of sarcopenia defined by different criteria

In the whole study population, the prevalences of EWGSOP-defined, EWGSOP2-defined, modified EWGSOP-defined, and modified EWGSOP2-defined sarcopenia were 27.3%, 26.8%, 11.7%, and 9.9%, respectively (Table 1). Regardless of the definition of sarcopenia, the prevalence of sarcopenia was significantly higher in the faller group than in the non-faller group. Similarly, the prevalence of sarcopenia was significantly higher in individuals with at least one hospitalization than in individuals without hospitalization during the follow-up (Table 1).

The prevalences of EWGSOP-defined, EWGSOP2-defined, modified EWGSOP-defined, and modified EWGSOP2-defined sarcopenia in the follow-up group and in the lost to follow-up group were 26.8% versus 42.9% (p = 0.185), 26.2% versus 42.9% (p = 0.168), 11.4% versus 21.4% (p = 0.250), and 10.0% versus 7.1% (p = 0.725), respectively.

### Sarcopenia and the incidence of falls

During the one-year follow-up, 98 participants (26.5%) had at least one fall. Regardless of the definitions, the prevalence of sarcopenia was significantly higher in the faller group than in the nonfaller group (Table 1). Modified EWGSOP2-defined sarcopenia appeared to be better than the other three sarcopenia definitions for predicting the incidence of falls (Supplementary Table 2), but the difference between these sarcopenia definitions was not statistically significant.

Table 2 shows the results of Cox proportional hazard models of different sarcopenia definitions for predicting the incidence of falls and hospitalization. After multivariable adjustment, EWGSOP2-defined sarcopenia (HR 1.86, 95% CI 1.22–1.84) and modified EWGSOP2-defined sarcopenia (HR 2.09, 95% CI 1.23–3.55) were significantly associated with an increased incidence of falls. Although EWGSOP-defined sarcopenia and modified EWGSOP-defined sarcopenia also have a trend to be associated with the incidence of falls, the results were not statistically significant (Table 2).

**Table 2:** Different sarcopenia definitions for predicting the incidence of falls or hospitalization according to Cox Regression Models.

	Unadjusted	Model 1	Model 2
**Incidence of falls**			
EWGSOP-defined sarcopenia	1.90 (1.27–2.86)	1.52 (0.99–2.34)	1.51 (0.98–2.33) ^a^
EWGSOP2-defined sarcopenia	2.39 (1.60–3.56)	1.86 (1.22–2.83)	1.86 (1.22–2.84) ^a^
Modified EWGSOP-defined sarcopenia	2.35 (1.44–3.85)	1.69 (1.01–2.82)	1.65 (0.98–2.79) ^a^
Modified EWGSOP2-defined sarcopenia	2.83 (1.73–4.64)	2.07 (1.23–3.46)	2.09 (1.23–3.55) ^a^
**Incidence of hospitalization**			
EWGSOP-defined sarcopenia	1.84 (1.04–3.27)	1.51 (0.81–2.77)	1.48 (0.81–2.72) ^b^
EWGSOP2-defined sarcopenia	1.88 (1.07–3.30)	1.56 (0.87–2.82)	1.57 (0.87–2.83) ^b^
Modified EWGSOP-defined sarcopenia	2.31 (1.19–4.51)	1.87 (0.93–3.77)	1.98 (0.98–3.98) ^b^
Modified EWGSOP2-defined sarcopenia	2.36 (1.18–4.72)	1.92 (0.94–3.94)	2.07 (1.01–4.27) ^b^

Data are presented as hazard ratios (95% confidential intervals).

Model 1: adjusted for age and gender.

^a.^Model 2: adjusted for age, gender, coronary heart disease, cognitive impairment, and history of falls.

^b.^Model 2: adjusted for age, gender, and hypertension.

EWGSOP: European Working Group on Sarcopenia in Older People; EWGSOP2: the updated version of the European Working Group on Sarcopenia in Older People.

**Figure 1:**
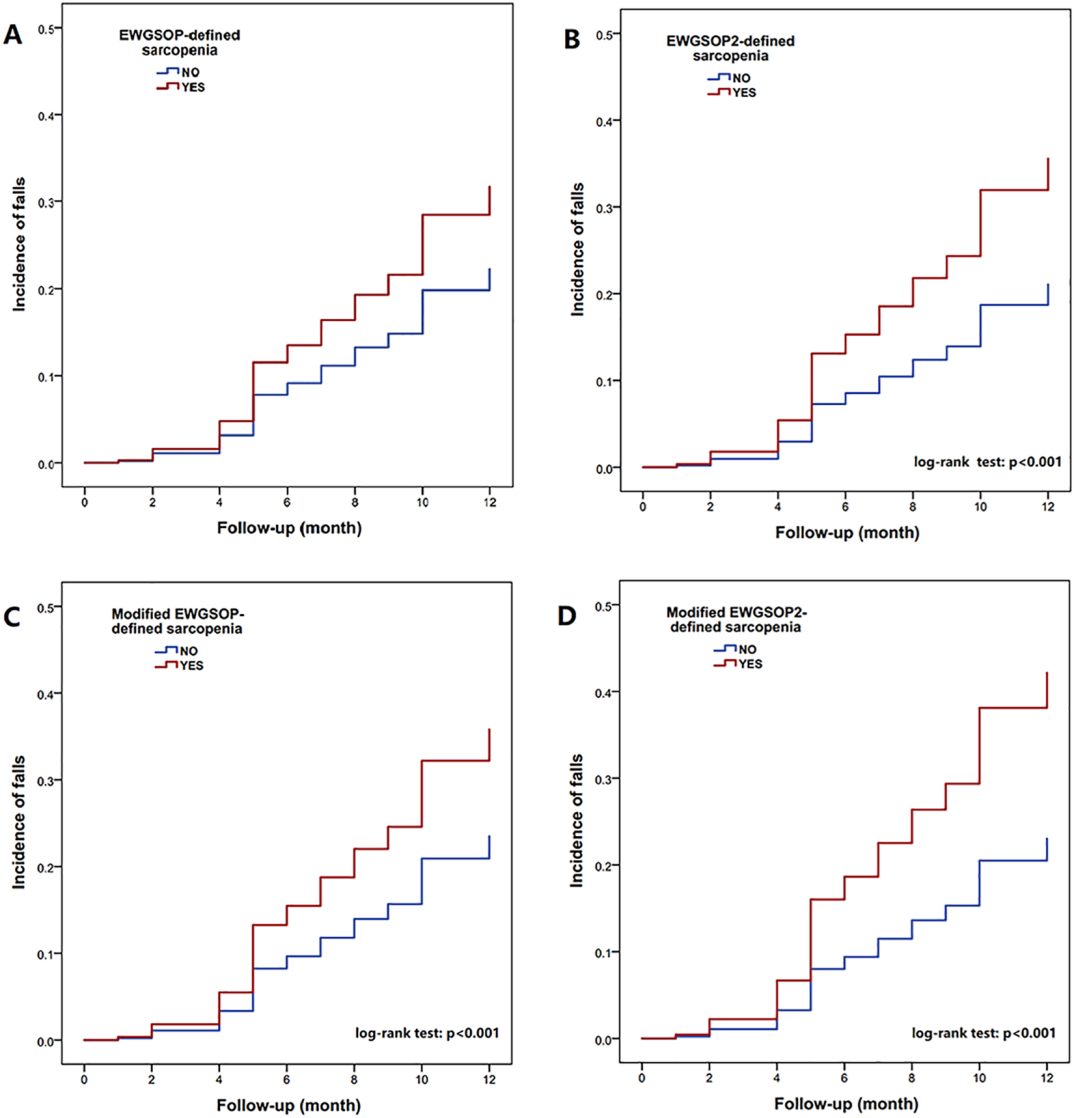
The Kaplan–Meier curves of different sarcopenia definitions for predicting the incidence of self-reported falls during the one-year follow-up.

The Kaplan–Meier curves of different sarcopenia definitions for predicting the incidence of falls during the one-year follow-up are shown in Fig. 1.

### Sarcopenia and the incidence of hospitalization

Fifty-one participants (13.8%) were hospitalized at least one time during the follow-up. Regardless of the definitions, the prevalence of sarcopenia was significantly higher in the hospitalization group than in the nonhospitalization group (Table 1). Modified EWGSOP2-defined sarcopenia seemed to be better than the other three sarcopenia definitions for predicting the incidence of hospitalization, but the difference between these sarcopenia definitions was also not significant (Supplementary Table 2).

**Figure 2:**
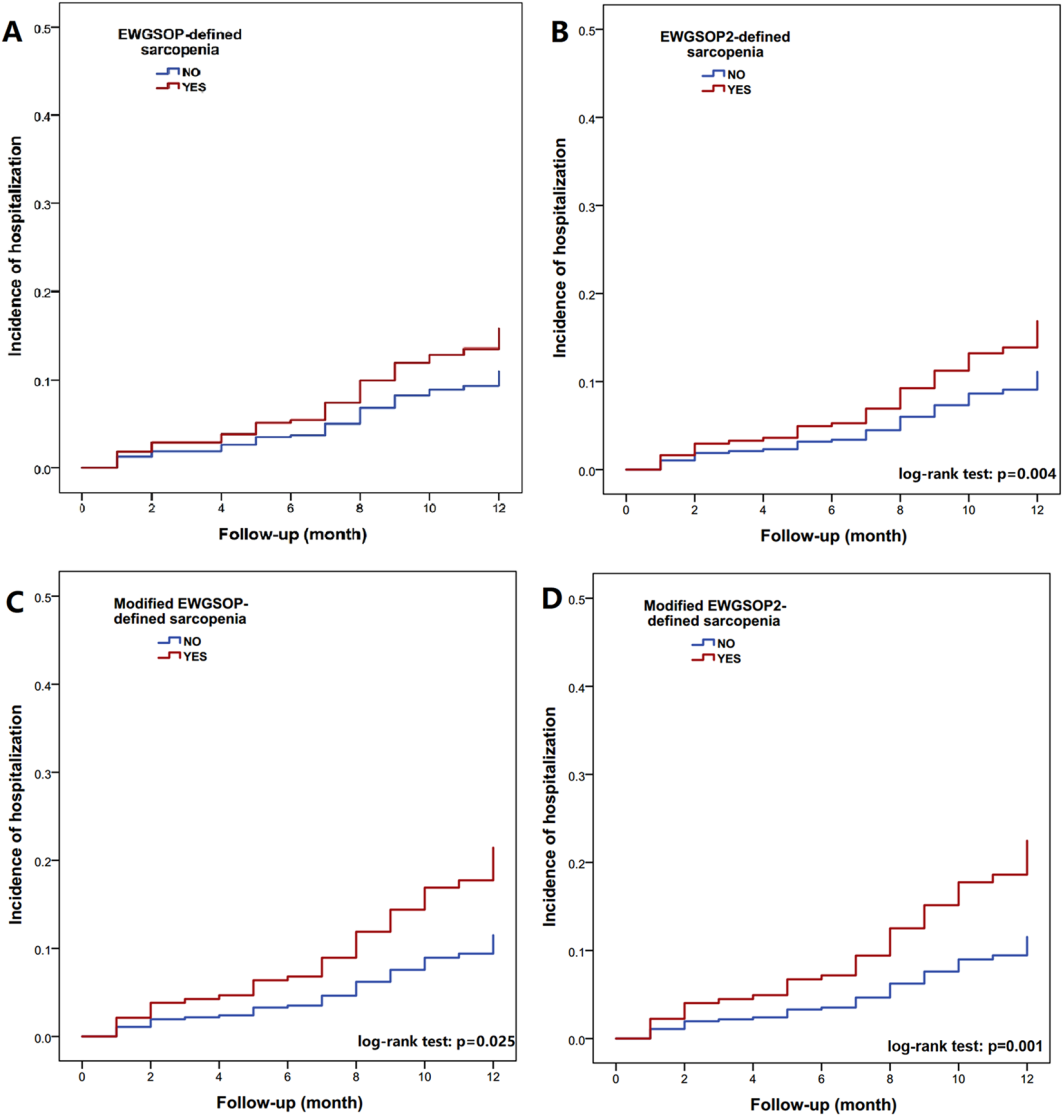
The Kaplan–Meier curves of different sarcopenia definitions for predicting the incidence of hospitalization during the one-year follow-up.

After multivariable adjustment, only modified EWGSOP2-defined sarcopenia (HR 2.07, 95% CI 1.01–4.27) was significantly related to an increased incidence of hospitalization, although EWGSOP-defined, modified EWGSOP-defined, and EWGSOP2-defined sarcopenia also have a trend to be associated with the incidence of hospitalization (Table 2). The Kaplan–Meier curves of different sarcopenia definitions for predicting the incidence of hospitalization during a one-year follow-up are shown in Fig. 2.

## Discussion

Our study demonstrated that EWGSOP2-defined sarcopenia appears to be more sensitive than EWGSOP-defined sarcopenia for predicting the incidence of falls in community-dwelling older adults. The modification of the cut-off points of the components of sarcopenia according to the characteristics of the study population may further improve the predictive value of EWGSOP2-defined sarcopenia for the incidence of falls.

Falls in older adults are a major cause of injury that may result in fracture, disability, poor quality of life, and death^18^. Some studies have been conducted to explore the possible association between sarcopenia and the incidence of falls^19,20^, fall risk^21,22^, previous falls^23^, recurrent falling^24^, fall-related hospitalization^25^, and fall-related injuries^26^ among older adults. However, when focusing on the association between sarcopenia and the incidence of falls, the evidence in the literature is limited. A prospective study reported that EWGSOP-defined sarcopenia was significantly associated with the three-year incidence of falls among community-dwelling older adults^19^. Another prospective study found a similar result in a population of community-dwelling people aged 80 years or older during a two-year follow-up^20^. Notably, both studies were conducted in Caucasian populations.

Most recently, the newly published International Clinical Practice Guidelines for Sarcopenia (ICFSR) recommend that “clinical trials also need to focus on outcomes relevant to stakeholders, clinicians, and patients”, such as “…, the rate of falls, …”^27^. Our study followed the recommendation of the ICFSR guideline. Our study showed that EWGSOP-defined sarcopenia could not predict the one-year incidence of falls, even after modifying the cut-off points of the sarcopenia components according to the characteristics of our study population (i.e., the modified EWGSOP). However, both EWGSOP2-defined and modified EWGSOP2-defined sarcopenia were predictors of the incidence of falls even after adjustment for a history of falls and other confounding factors. On the other hand, it is noteworthy that the ICFSR recommends the use of DXA to assess body composition; however, we applied a BIA device to estimate body composition in this study, which might overestimate the ASM of our participants.

A recent systematic review of five prospective studies concluded that sarcopenia was a significant predictor of hospitalization among older adults^28^. However, only two prospective studies were conducted in community-dwelling older adults and their results were conflicting^29,30^. Cawthon and colleagues^29^ studied sarcopenia and hospitalization in community-dwelling older men. These authors defined sarcopenia according to five sarcopenia definitions including the EWGSOP and they concluded that sarcopenia was not associated with hospitalization regardless of the sarcopenia definitions^29^. In contrast, Bianchi and colleagues^30^ reported that EWGSOP-defined sarcopenia was a significant predictor of hospitalization. In our study population, EWGSOP-defined, modified EWGSOP-defined, and EWGSOP2-defined sarcopenia failed to predict hospitalization, but modified EWGSOP2-defined sarcopenia was a predictor of hospitalization. This finding deserves further validation in the future.

Interestingly, the prevalences of EWGSOP-defined and EWGSOP2-defined sarcopenia were very similar in our study population no matter using the original cutoffs (27.3% vs. 26.8%) or the cutoffs modified according to the characteristic of our study population (11.7% vs. 9.9%). In the EWGSOP2, individuals with a low SMM and a low GS (but with a normal HS) were no longer considered as having sarcopenia (Supplementary Table 1). Our finding implies that the exclusion of these individuals may not significantly influence the diagnosis of sarcopenia and these individuals appear to be less likely to fall compared to those with a low HS. Previous studies also demonstrated that a low HS was the independent risk factor of fall episodes in older adults^31,32^. However, further studies are required to determine whether these individuals (with a low SMM and a low GS but a normal HS) should be considered having sarcopenia or not.

Our study has several limitations. First, the observational nature of our study means that the causality between sarcopenia and falls (or hospitalization) cannot be established. Second, we did not collect information regarding some potential confounders, such as lifestyle factors, physical frailty, nutrition status, and medication use, and some important outcomes, such as the reasons for hospitalization. Third, we did not perform subgroup analysis based on gender because of the relatively small sample size of our study population, especially men. However, we adjusted for gender in Cox proportional hazard models and gender was not a significant predictor of either falls or hospitalization in these models. Fourth, because the EWGSOP did not provide recommend cutoff values of ASMI measured by BIA, we had to apply the EWGSOP cutoff values of ASMI measured by DXA (instead of BIA). This might induce bias to our results as we estimated the ASMI using BIA. Last, the generalizability of our results may be limited to Chinese community-dwelling older adults, especially the results regarding the modified EWGSOP and modified EWGSOP2. Therefore, our results need to be validated in multiple cohorts.

## Conclusions

The prevalences of EWGSOP-defined and EWGSOP2-defined sarcopenia were very similar no matter using the original cutoffs or the cutoffs modified according to the characteristics of our study population. EWGSOP2-defined sarcopenia is better than EWGSOP-defined sarcopenia for predicting the one-year incidence of falls or hospitalization, especially when using the modified cutoffs. Our study preliminarily validates the predictive value of EWGSOP2-defined sarcopenia for falls in Chinese community-dwelling older adults. Our study also implies that it might be necessary to modify the cut-off points of the EWGSOP2 when applying it in Chinese older populations. However, more prospective studies in different ethnic populations are warranted to validate these results and to evaluate the value of the EWGSOP2 for other important outcomes, such as quality of life and mortality.

## Supplementary information

Supplementary Tables

## Data Availability

The datasets generated during and/or analyzed during the current study are available from the corresponding author on reasonable request.
