# Supplementary material for: Sarcopenia for predicting falls and hospitalization in community-dwelling older adults: EWGSOP versus EWGSOP2
Source: Sci Rep. 2019 Nov 27;9:17636. doi: 10.1038/s41598-019-53522-6 (PMC6881315; doi:10.1038/s41598-019-53522-6)
Supplement: Supplementary file 1 — Supplementary Tables [file 41598_2019_53522_MOESM1_ESM.pdf]

Title Page

Title:

Sarcopenia for predicting falls and hospitalization in community-dwelling older adults: EWGSOP versus EWGSOP2

Names and institutions of all the contributing authors:

Ming Yang [1,2#], Ying Liu [1#], Yun Zuo[3], Huairong Tang[4*]

1. The Center of Gerontology and Geriatrics, West China Hospital, Sichuan University, No. 37 Guoxue Lane, Chengdu, Sichuan, China
2. Precision Medicine Research Center, West China Hospital, Sichuan University, No. 37 Guoxue Lane, Chengdu, Sichuan, China
3. Health Management Center, Shangjin Nanfu Hospital, Chengdu, Sichuan, China
4. Health Management Center, West China Hospital, Sichuan University, No. 37 Guoxue Lane, Chengdu, Sichuan, China

#. Ming Yang and Ying Liu contribute equally to this work.

Corresponding author:

Huairong Tang, Professor

Phone: +86 28 85422131

Fax: +86 28 85422131

Email: 1651682099@qq.com

Supplementary Table 1. The diagnostic criteria for sarcopenia in this study

	① Low muscle mass	② Low HS	③ Low GS	Diagnostic criteria
EWGSOP	ASMI < 7.26 kg/m^2 for men;	<30 kg for men;	<0.8 m/s for both gender	① + ② or ① + ③
	ASMI < 5.5 kg/m^2 for women	<20 kg for women		
EWGSOP2	ASMI < 7.0 kg/m^2 for men;	<27 kg for men;	-	① + ②
	ASMI < 6.0 kg/m^2 for women	<16 kg for women		
Modified EWGSOP	ASMI < 6.28 kg/m^2 for men;	<22.2 kg for men;	<0.8 m/s for both gender†	① + ② or ① + ③
	ASMI < 5.08 kg/m^2 for women †	<14 kg for women†		
Modified EWGSOP2	ASMI < 6.28 kg/m^2 for men;	<22.2 kg for men;	-	① + ②
	ASMI < 5.08 kg/m^2 for women †	<14 kg for women†		

ASMI, appendicular skeletal muscle index; EWGSOP, European Working Group on Sarcopenia in Older People; EWGSOP2: the updated version of the European Working Group on Sarcopenia in Older People; HS, handgrip strength; GS, gait speed.

† The cut-off points were based on the lowest quintile values of the distribution of our study population (the lowest quintile value of GS in our study population was 0.8 m/s, which was the same as the EWGSOP and EWGSOP2 recommendations).

Supplementary Table 2. Different sarcopenia definitions for predicting the incidence of falls and hospitalization according to the receiver operating curve models

	AUC*	SE	95% CI
**Incidence of falls**			
EWGSOP-defined sarcopenia	0.559	0.0273	0.507 - 0.611
EWGSOP2-defined sarcopenia	0.571	0.0217	0.519 - 0.622
Modified EWGSOP-defined sarcopenia	0.584	0.0292	0.532 - 0.635
Modified EWGSOP2-defined sarcopenia	0.613	0.0279	0.561 - 0.663
**Incidence of hospitalization**			
EWGSOP-defined sarcopenia	0.562	0.0368	0.510 - 0.613
EWGSOP2-defined sarcopenia	0.572	0.0311	0.520 - 0.623
Modified EWGSOP-defined sarcopenia	0.577	0.0383	0.525 - 0.628
Modified EWGSOP2-defined sarcopenia	0.596	0.0376	0.544 - 0.646

AUC, area under the curve; CI: confidence interval; SE: standard error

* According to the Delong method, there was no significant difference between the AUCs of the four ROC curves with each other.
